# Customized a Ti6Al4V Bone Plate for Complex Pelvic Fracture by Selective Laser Melting

**DOI:** 10.3390/ma10010035

**Published:** 2017-01-04

**Authors:** Di Wang, Yimeng Wang, Shibiao Wu, Hui Lin, Yongqiang Yang, Shicai Fan, Cheng Gu, Jianhua Wang, Changhui Song

**Affiliations:** 1 School of Mechanical and Automotive Engineering, South China University of Technology, Guangzhou 510640, China; scut061389@163.com (D.W.); ym_zwang@163.com (Y.W.); siberghost@126.com (S.W.); linhui_zj@163.com (H.L.); song_changhui@163.com (C.S.); 2 The Third Affiliated Hospital of Southern Medical University, Guangzhou 510600, China; fanscyi@sohu.com (S.F.); 15622127207@163.com (C.G.); 3 Hospital of Orthopedics, Guangzhou General Hospital of Guangzhou Military Command, Guangzhou 510010, China; jianhuawangddrr@163.com

**Keywords:** metal additive manufacturing, selective laser melting, bone plate, pelvic fracture, Ti-6Al-4V

## Abstract

In pelvic fracture operations, bone plate shaping is challenging and the operation time is long. To address this issue, a customized bone plate was designed and produced using selective laser melting (SLM) technology. The key steps of this study included designing the customized bone plate, metal 3D printing, vacuum heat treatment, surface post-processing, operation rehearsal, and clinical application and evaluation. The joint surface of the bone plate was placed upwards with respect to the build platform to keep it away from the support and to improve the quality of the joint surface. Heat conduction was enhanced by adding a cone-type support beneath the bone plate to prevent low-quality fabrication due to poor heat conductivity of the Ti-6Al-4V powder. The residual stress was eliminated by exposing the SLM-fabricated titanium-alloy bone plate to a vacuum heat treatment. Results indicated that the bone plate has a hardness of HV1 360–HV1 390, an ultimate tensile strength of 1000–1100 MPa, yield strength of 900–950 MPa, and an elongation of 8%–10%. Pre-operative experiments and operation rehearsal were performed using the customized bone plate and the ABC-made pelvic model. Finally, the customized bone plate was clinically applied. The intraoperative C-arm and postoperative X-ray imaging results indicated that the customized bone plate matched well to the damaged pelvis. The customized bone plate fixed the broken bone and guides pelvis restoration while reducing operation time to about two hours. The customized bone plate eliminated the need for preoperative titanium plate pre-bending, thereby greatly reducing surgical wounds and operation time.

## 1. Introduction

Surgery assisted by both computer-aided design and 3D printing is a novel technological approach which has attracted increasing popularity amongst scholars in varied medical fields [1,2,3]. Currently, the application of 3D printing technology in medical science can be divided into three main branches: (1) anatomical models; (2) surgical instruments; and (3) implants and prostheses [4,5]. Highly accurate models fabricated using 3D printing can provide a complete anatomical replica of a patient’s organ or tissue defect, improving the prospects of helping doctors with disease diagnosis, design of pre-operative schemes, pre-operative exercise, etc. For instance, Condino et al. printed a complete abdominal model of their patients derived from CT images [6]; Tam et al. employed 3D printed models to help make operative plans for patients with large scapular osteochondroma complicating congenital diaphysealaclasia [7]. Additionally, 3D printed surgical instruments can play a guiding role in operations, improving surgical precision and reducing the length of operations. For example, Birnbaum et al. created customized polycarbonate templates using 3D printing, which enabled pedicle screws to be placed at preset locations during the operation, thus achieving precise and rapid positioning, thus considerably reducing operation time [8]. Finally, 3D printed medical implants can be customized specifically for each patient, which has gradually developed as a major application in recent years. For instance, Horn et al. repaired a bone lesion around patients’ noses using 3D printed titanium mesh implants [9], thus successfully repairing their appearance. Despite the gradual increased application of 3D printing in surgery, its use in pelvic fracture is limited to the fabrication of models of pelvises. For example, Niikura et al. conducted pre-operative pre-bending of conventional bone plates and estimated the position and length of screws through a 3D-printed pelvic model [10]. Metal 3D printing technology has been favorably demonstrated in the orthopedic field including fabricating customized knee joint prostheses, femoral prostheses, and spinal prostheses [11,12,13].

However, the therapy of complex pelvic fracture by fabrication of customized bone plates through 3D printing technology has not been introduced. Pelvic fracture results in high rates of both disability and fatality [14,15]. The current therapeutic method is to achieve anatomical reduction and compressive fixation with conventional bone plates [16,17]. This has the drawback that poorly-matched bone plates, with no pre-operative bending and having no bending at the pores, restrict the degree of attachment between the bond plate and surface. Adjustment of the bonding plate is then conducted according to doctors’ experience during the operation, leading to operations that are both difficult and lengthy [16,18].

Metal 3D printing allows the direct fabrication of functional parts with complex shapes from digital models. Many investigation had been carried out recently. Sing et al. summarized the current progress of two additive manufacturing (AM) processes suitable for metallic orthopaedic implant applications, namely, selective laser melting (SLM) and electron beam melting (EBM) [19], additive manufactured biomaterials, such as 316L stainless steel, titanium-6aluminium-4vanadium (Ti6Al4V) and cobalt-chromium were highlighted in their review. Do et al. studied the effect of laser energy input on the microstructure, physical, and mechanical properties of Ti-6Al-4V alloys by selective laser melting [20]; in their work the porosity/relative density, surface quality, microstructure, and mechanical properties were investigated on the selective laser melted Ti-6Al-4V alloy specimens fabricated with a wide range of laser energy inputs. Yadroitsev used an X-ray diffraction technique and numerical simulation for investigating the residual stress in SLM samples fabricated from 316L stainless steel and Ti6Al4V alloy [21]. 

The present study is aimed at investigating the requirements of complex pelvic fracture surgery. Computer-aided design and selective laser melting (SLM) technologies were employed to fabricate completely-attached, customized, titanium alloy bone plates for pelvic fracture to improve the quality of the fracture reduction and to reduce the length of the operation. The experiment also has to prove if the customized bone plate can meet mechanical property requirements for standard medical bone plates. Combined with optimizable pelvic fracture therapy using the lateral approach of the rectus abdominis, a minimally invasive, customized, and precise operation can truly be achieved. This technology enabled quick fabrication of a Ti-6Al-4V customized bone plate, fix the broken bone, and guide pelvis restoration, and its enormous advantages are proved by clinically testing.

## 2. Customized Design of Bone Plate for Pelvic Fracture

The design process used in the present study is based on 4 customized bone plates which have been successfully used to repair pelvic fractures. Specifically, a 53-year-old female patient who was involved in a car accident was diagnosed as having a left-side acetabular fracture (T type + antetheca).

The design procedure of customized bone plate is as follows.

### 2.1. Procedure A: Extraction of Computed Tomography (CT) Data of the Target Pelvis

**Figure 1:**
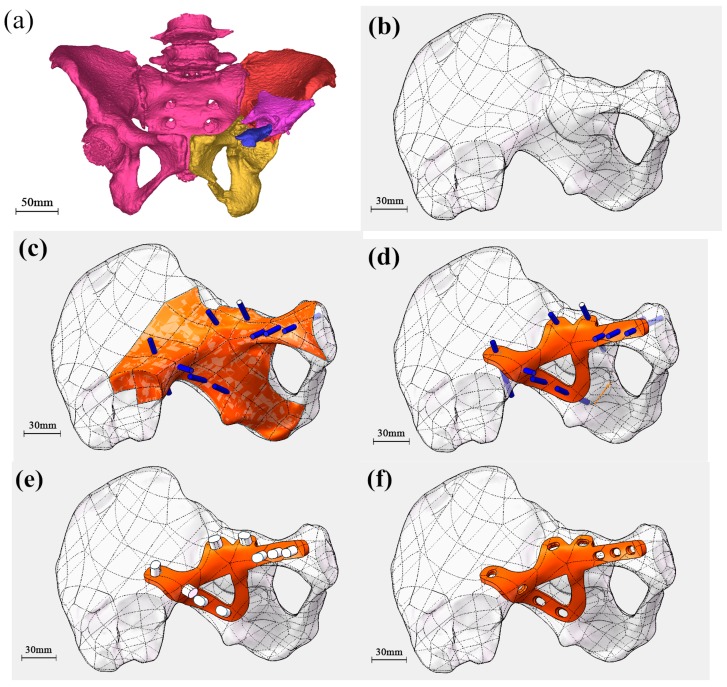
Key procedures in designing customized bone plate: (**a**) CT data model of pelvis; (**b**) Generation of pelvic model; (**c**) Fabrication of the initial shape; (**d**) Determination of the shape and thickness of the bone plate; (**e**) Modeling of the screw holes; and (**f**) The final bone plate model.

The CT data of the patient’s pelvis was imported into Mimics 16.0. The data of the patient’s unbroken side was selected and image processing was performed to obtain raw data of the broken side, which was saved as STL (binary) data. The CT data model is shown in Figure 1a.

### 2.2. Procedure B: Generation of the Pelvis Model

The STL data was imported into Geomagics software. Preliminary repair of this model and elimination of noise inside the bone was initially completed by “network doctor”, followed by the software algorithm “extract surface-construct patches-construct grids-surface fitting”, which created a precise surface function. The resultant model was finally saved as an STP AP203 formatted file, which was imported into Solidworks software to obtain a target pelvis model, as presented in Figure 1b.

### 2.3. Procedure C: Fabrication of the Original Shape of the Customized Bone Plate

Starting with the pelvic model image and taking the unbroken side as a reference, an approximate size range of the bone plate was estimated and these regions again extracted to serve as the initially designed surface. Determination of screw positions in some regions of the bone plate was conducted according to the degree of damage to the patient’s bone. The posterior column anterograde screw was employed to fix the acetabulum posterior column, which was screwed-in from the ilium surface and penetrated from the ischial tuberosity. The small screw acted as a fixed role after reduction of the broken pelvis, with sufficient thickness at the position of implantation, which was not the penetration position, and the direction of acetabular fossa could, therefore, be guaranteed, as presented in Figure 1c.

### 2.4. Procedure D: Determination of the Shape and Thickness of the Customized Bone Plate

Surface trimming was performed according to the screw positions so as to obtain regularity in the surface of the bone plate, which was thickened outwards. The bone plate was constructed with a thickness of 3–3.5 mm to ensure screw strength, as shown in Figure 1d.

### 2.5. Procedure E: Fabrication of Screw Holes of the Customized Bone Plate

The corresponding “punching instrument” was designed according to the shapes and specifications of the different screws. The surface was extracted and screw holes at the corresponding positions of the steel plate were added. M3.5 compressive screws and M6.5 pressure screws, which are generally used for conventional bone plates, were employed in the present study, as shown in Figure 1e.

### 2.6. Procedure F: Finishing of the Edge of Customized Bone Plate

Finishing was performed on the edge of customized bone plate. The boundary was smoothed to obtain the final bone plate model, as presented in Figure 1f. The model was saved as an STL file and then exported.

The rationale for the design of the plate is based on the requirements of the clinical doctor. He will decide the bone and plate contact surface size, and all other detailed dimensions as per his/her clinical experience. The design of the guider should also consider the restriction of the SLM process, such as the thin wall’s size should not be too small, the contact surface’s quality should be guaranteed, and the plate’s thickness should be thick enough to maintain the strength in clinical use. The design requirements include: (1) the joint surface should completely fit the patient’s bone surface; (2) the strength of the customized plate should be the same or more than the traditionally-made plate; (3) the key positions, such as the screw holes, should be of sufficient accuracy.

## 3. Experimental Methods

### 3.1. Processing Optimization of Metal 3D Printing Devices and Materials

**Figure 2:**
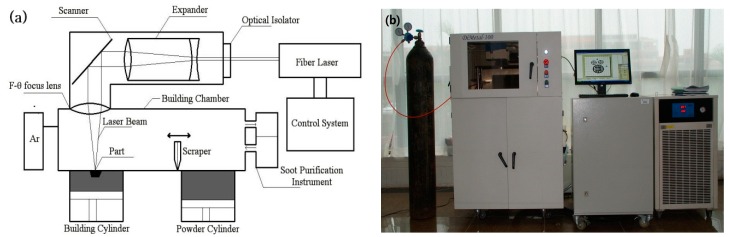
Principle of SLM manufacturing and the experimental DiMetal-100 equipment. (**a**) Principle of SLM manufacturing; and (**b**) DiMetal-100.

The experiments were carried out using a self-developed selective laser melting (SLM) machine, DiMetal-100 (SCUT, Guangzhou, China). Figure 2a shows the principle of SLM manufacturing and the DiMetal-100 equipment used is shown in Figure 2b. The setup consisted of a fiber laser, optical-path transmission unit, sealing chamber (including the powder recoating device), mechanical drive, and controlling systems, as well as the processing software. The laser was directed using a scanning galvanometer, which was then focused through the f-θ lens, and melts the metallic powders on the plane selectively, followed by stacking them layer-wise into metal parts. The machine has a scanning speed in the range of 10–5000 mm/s, thickness of the processing layer in the range of 20–100 µm, and a laser focusing spot diameter of 70 µm. The largest size of the part produced was 100 mm × 100 mm × 120 mm. Since the powder was fully melted during the process, protection of the SLM-processed parts from oxidation was essential. Therefore, processing of all of the metal powders was carried out in an argon or nitrogen atmosphere, with not more than 0.15% O_2_.

**Figure 3:**
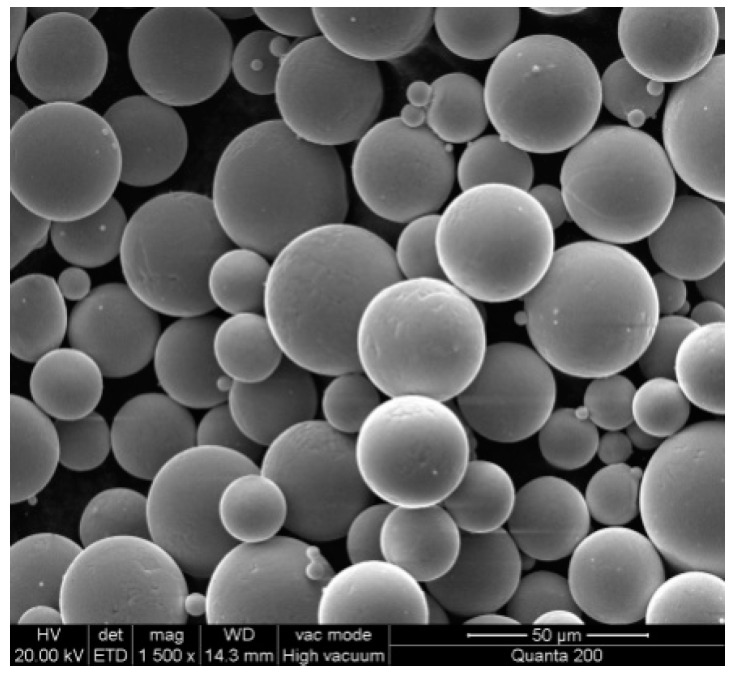
The SEM image of Ti6Al4V powder having the size of 500 mesh.

Ti-6Al-4V powder, fabricated by the Falcon Tech Co., Ltd. (Wuxi, China), was used for fabrication to satisfy the requirements of ASTM F2924 [22]. Its composition is listed in detail in Table 1. The mean powder particle diameter was measured as 36 μm. A 1500× magnification image obtained through scanning electron microscopy (SEM, Nova NanoSEM 430, FEI company, Hillsboro, OR, USA) is shown in Figure 3.

**Figure 4:**
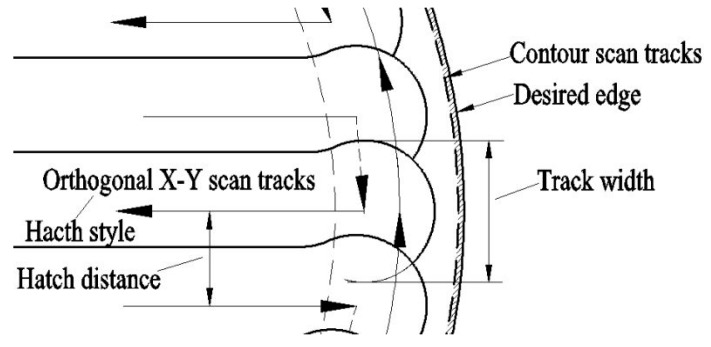
Schematic diagram of the laser scanning and track overlapping.

The optimized process was obtained through multiple technological optimizations. First, the laser spot diameter was set to be 70 µm and unchangeable, and orthogonal experiments were designed to determine the laser power, scanning speed, layer thickness, scanning spacing, and the target, in order to obtain dense and high-surface-quality parts. In order to describe more definitely, a schematic diagram (Figure 4) is applied to explain these parameters. Track width is much related to laser spot diameter and energy input; scanning spacing is the offset between two adjacent tracks; hatch style (scanning strategy) is the laser scanning direction in one layer and between adjacent layers, and it significantly affects the accumulated stress; hatch overlap is determined by scanning spacing and track width, and it is usually set around 30%. Then, further experiments were designed to determine the exact laser energy input by changing the laser power and scanning speed. The optimized SLM fabricating parameters of the Ti6Al4V powder are shown in Table 2.

### 3.2. Experimental Procedure

**Figure 5:**
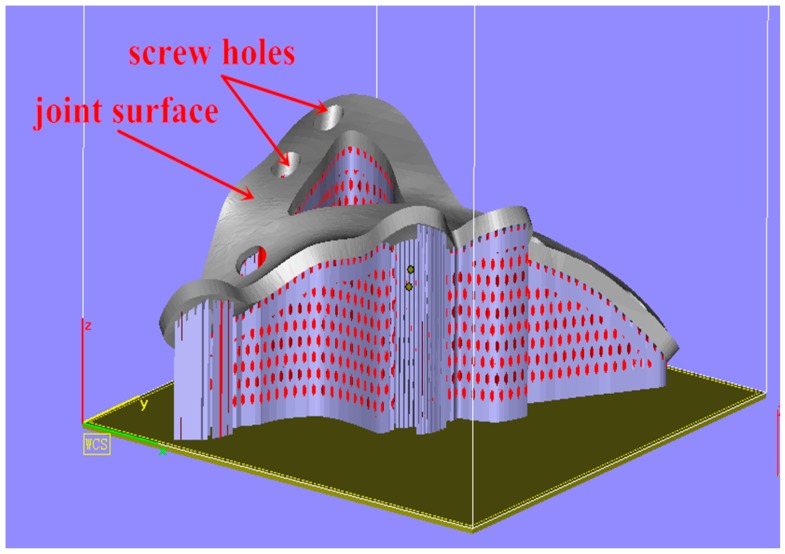
Orientation placement of the customized bone plate and addition of support (Magics 16.0).

First, a customized bone plate for a complex pelvic fracture was designed with the guidance of an orthopedic surgeon, which was then saved as an STL file and exported; the as-designed customized bone plate file in STL format was imported into Magics 16.0. The customized bone plate was then placed according to spatial orientation. Specifically, the attached face between the bone plate and bone was placed upwards to guarantee a low degree surface roughness; support was added to the file of the oriented, placed, customized bone plate, as presented in Figure 5. Typically, top and bottom surfaces are poor, the side surfaces are better due to the layer slicing, and the bottom surface will be post-processed before anodizing.

Then parameters, including radius compensation and layer thickness of each slice were set in the Magics 16.0 software to conduct slicing, after which the CLI formatted file was obtained and imported into a self-developed laser path planning software. Path planning for the scanning model of the different files was then performed; afterwards the processed data were imported into a self-developed DiMetal-100 metal 3D printing device to process.

The next step is heat treatment. This process was conducted in a vacuum, and performed on the printed customized bone plate with processing details as follows: the sample was heated to 820 °C over 3 h, incubated for 2 h, then cooled to 450 °C in a furnace, after which the furnace was opened and the sample air-cooled. After the heat treatment, post-treatment of the surface of the customized bone plate following heat treatment was performed. After heat treatment the part will be removed from the base plate by wire-electrode cutting, then the support structure will be removed from the part by hand. The surface treatment procedures included roll cast, oil cleaning, acid pickling, polishing, anodizing, cleaning, and disinfection were performed, after which various properties of the customized bone plate were evaluated.

Finally, the pelvic model of the customized bone plate and ABS material was matched to perform operation rehearsal. Subsequently, the customized bone plate was used in surgery, the operation evaluated by C-arm throughout surgery, and by X-ray imaging afterwards.

### 3.3. Test Methods

The SLM-built parts’ hardness were tested using a digital micro-hardness HVS-1000 apparatus (Shunhua, Shenzhen, China). The measurement resolution of the HVS-1000 was 0.01 μm, with a measurement range of 1–3000 HV. The ultimate tensile strength of the manufactured part using SLM was tested using an electronic universal testing machine, CMT5105 (MTS, Eden Prairie, MN, USA). The CMT5105 has a relative error value of the test force of ±0.5%, the resolution of the test force was 1/300,000 FS, the relative error value of the deformation was ±0.50%, and the displacement resolution was 1 µm.

## 4. Results and Discussion

### 4.1. SLM Processing and Heat Treatment of Customized Bone Plate

**Figure 6:**
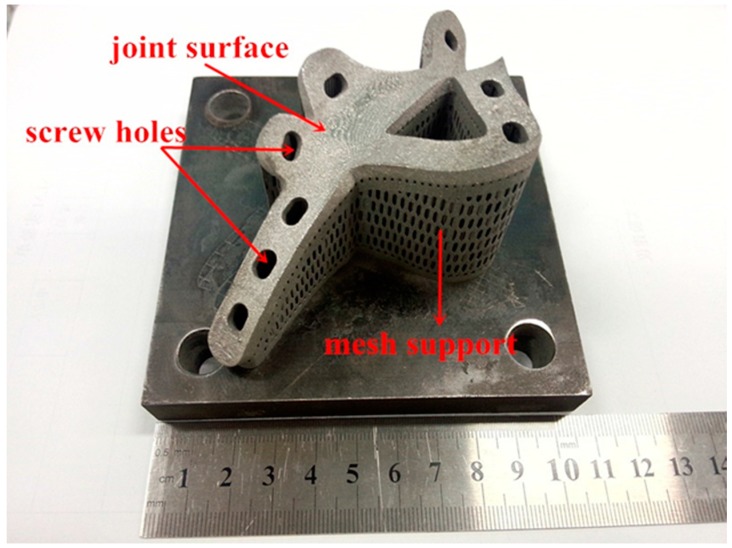
Customized bone plate after SLM fabrication.

Figure 6 shows the final product of the printed part. Evidently, a bright metallic luster can be found at the surface of SLM fabricating part, with no obvious pits or deformation defects, thus demonstrating favorable fabricating quality.

During processing, in order to guarantee the effectiveness of the attachment of the customized bone plate to the complex surface, the joint surface of the bone plate was placed upwards to separate it from the support to improve the quality of the joint surface. Additionally, heat conduction was enhanced during the SLM fabrication process by adding a cone-type support beneath the bone plate to prevent low-quality fabrication due to poor heat conductivity of the Ti-6Al-4V powder.

The build orientation is of great importance to the SLM part’s quality, especially the surface quality. The build orientation was determined by the surface needs and surface quality. Here, the supporting structures should be avoided, and it should be determined by the production time. In this study, the orientation was very close to the best, because the upper surface (contact surface) quality should be guaranteed, so the contact surface could join firmly with the bone surface.

### 4.2. Measurement of Mechanical Properties of Customized Bone Plate

It is generally recognized that both the hardness and ultimate tensile strength of metal 3D-printed parts are greater than that of ordinary cast parts, close to the mechanical properties of forged parts, while having low elongation [23,24]. In addition, a large residual stress can occur in the fabricating process of Ti-6Al-4V, and so heat treatment for the SLM molded customized bone plate should, thus, be conducted. The processing details are as follows: the sample was heated to 840 °C over a period of 3 h and incubated for 2 h, then cooled to 450 °C in a furnace, after which the door was opened and the sample air-cooled. The mechanical properties of 3D printed part are listed in Table 3. The hardness of the customized bone plate after heat treatment was determined to be HV1 360–390, which was higher than that of a conventional cast part (HV1 320). A possible reason may be that rapid melting and solidification are involved in metal 3D printing, thus resulting in the formation of small grains. The effect of fine-grain strengthening would contribute to its greater hardness. The ultimate tensile strength, yield strength, and elongation of the 3D-printed part following heat treatment were measured to be 1000–1100 MPa, 900–950 MPa and 8%–10%, respectively, thus satisfying the mechanical requirements of conventional titanium alloy [25].

### 4.3. Pre-Operative Simulation and Clinical Application

#### 4.3.1. Surface Treatment and Pre-Operative Simulation

**Figure 7:**
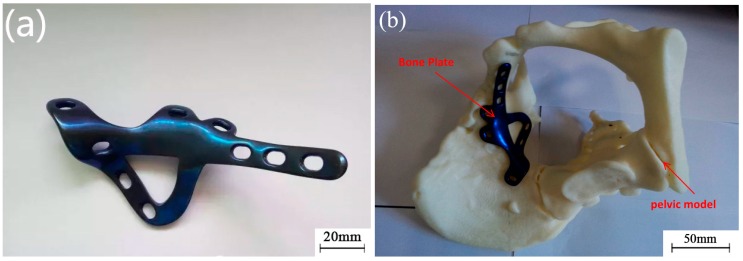
Surface treatment and pre-operative simulation: (**a**) Customized bone plate after surface post-treatment; and (**b**) The match between bone plate and pelvic model.

Anodizing was conducted for the customized bone plate following heat treatment to prevent any potential threat of poisonous metal ions leaching into the patient’s body and to obtain a bone plate which fulfilled the standard of a surgical implant, as presented in Figure 7a.

The pelvic model was printed using a Dimension SST 1200es FDM3D printer (Strasystem Company, Eden Prairie, MN, USA). ABS was employed as a material for printing the model with an accuracy of 0.1–0.2 mm, after which the customized bone plate, following surface treatment, was attached. The attaching effect between the customized bone plate and pelvic model was determined, as shown in Figure 7b. Apparently, the bone plate and pelvic model were perfectly matched, allowing a high degree of attachment contact. This also helps the doctors with simulated pre-operative exercise on the position of the pelvic fracture and predicting what may occur during the operation, thus reducing operation time and improving the surgical outcome.

#### 4.3.2. Clinical Application of Customized Bone Plate

**Figure 8:**
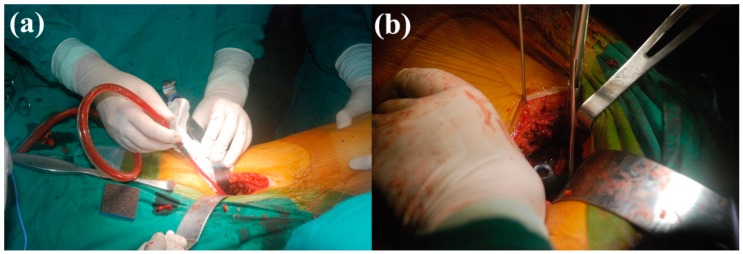
Clinical operation of the 3D-printed pelvic bone plate. (**a**) The first stage of the surgery; (**b**) The placement of the customized Bone Plate.

**Figure 9:**
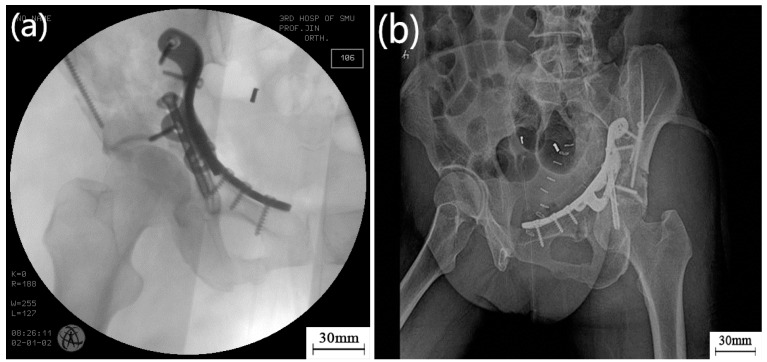
Examination of fixation effect of the bone plate during and after the operation: (**a**) C-arm image during operation; and (**b**) X-ray image following surgery.

Use in the clinic was approved by the Hospital Ethics Committees, and usage of the 3D-printed plate followed patient informed consent. Prior to use in surgery, high-temperature sterilization was performed on each customized bone plate. Figure 8 shows the clinical operation of the 3D-printed pelvic bone plate. The operation adopted lateral approach of the rectus abdominis, with an incision of only 7 cm and operating time of 2 h, a great reduction (4 h is required for a conventional bone plate operation), with bleeding of approximately 400 mL. Figure 9a shows the C-arm image during the operation, with the well-matched customized bone plate and fracture block visible. Figure 9b shows the CT reexamination image following surgery, where it can be deduced that the pelvic fracture presented favorable recovery and was, therefore, successful.

### 4.4. Discussion

According to the complete case reported above, utilization of an SLM-fabricated, customized bone plate demonstrated some advantages in its clinical application, which are as follows:

The first advantage is that only one customized bone plate was required to obtain a reduction of both the large and small broken bones for all patients, thus reducing the number of implants for each patient. Meanwhile, the incision required for the operation was minimized, and so less intra operative variation and improved accuracy could also be achieved.

The second advantage due to the shape was designed according to the initial pelvic model of the patient, thus the customized bone plate guided the restoration of the pelvis by the doctors, which greatly reduced operation time.

The next advantage is that since the customized bone plate was fabricated based on the varying nature of the fractures of the different patients, which were well matched with the fracture block, no intra-operative bending was involved. This method of therapy, combined with the lateral approach of the rectus abdominis, demonstrated significant advantages in minimally-invasive therapy of pelvic fracture, thus achieving “customized, precise, and minimally invasive” therapy.

Following completion of the first clinical application, our researchers recognized some problems in the design and the SLM fabrication process, which we have been able to optimize and improve.

#### 4.4.1. Improved Design of Customized Bone Plate

Based on the customized bone plate in this experiment, the design process and metal 3D printing technology required some improvements. The details of the improvements are as follows:

The first improvement is that a larger volume of the customized bone plate would result in a larger intra-operative wound, which would be more harmful to the patient. In the present experiment, the surface width and thickness were selected to be approximately 10 mm and 3 mm, respectively. Currently, a decrease in both width and thickness, while satisfying operating requirements, was taken into consideration.

The next improvement is because the bone plate did not serve as the main bearing material, a porous and light-weight structure could be introduced for the design of the bone plate if operational performance could be guaranteed [26], reducing the weight and cost of the customized bone plate, and be beneficial for the self-healing of the pelvis.

Afterwards, as the user of the bone plate, the surgeons have the final decision on the design and optimization of the bone plate. However, usually the surgeons lack expertise in the customization of complex curved surfaces. In addition to this, an experienced designer must tailor geometrical sizes of the parts as per the needs of the surgeons, such as the height of the bone plate, and location of the screw holes. Finally, the designers should take into account the limitations of principles in 3D metal printing. For example, the surface roughness of the 3D metal printed parts is inferior to the traditional method. The vertical hole fabricated through 3D metal printing has adherent dross or blockage. The resolution of the thin parts and tiny cylinders fabricated through 3D metal printing cannot be less than the width of a single melting track. Therefore, fabricating a high-precision metal bone plate that is suitable for clinical application requires synergy between doctors, designers, and 3D printing engineers.

#### 4.4.2. Improvement of SLM Fabrication Processing of a Customized Bone Plate

The fabrication process of the metal 3D printed bone plate also required improvement, detailed as follows:

The first improvement focuses on the support parameters. The customized bone plate is a large-sized part and presents a complex spatial structure, because the addition of a support is complicated. The primary requirement for the additional support is that it must be easy to remove without affecting the resultant fabricating. As the height and volume of the bone plate employed in this experiment were large and the addition of support was intensive, the selection of the support type and density needed optimization. In addition, as mentioned above, a cone-type support was needed because of poor heat conductivity, which would contribute to high support strength and which would, therefore, prolong the subsequent polishing time. Therefore, prior to the processing of customized bone plates of subsequent cases, the parameters of the support structure including tooth spacing and width, were optimized.

The second improvement is the heat treatment processing. Currently, it is generally acknowledged that the advantage of 3D-printed titanium alloy is its superior hardness and strength, whilst having the drawbacks of poor elongation and fatigue properties [27]. The heat treatment processing adopted in the present experiment mainly focused on elimination of the residual stress inside the part. Elongation was improved when both hardness and strength were guaranteed. In terms of the present effect, the elongation of the fabricated parts was lower than that of conventional processing part.

After optimization of the relevant procedures, at least four days were required from hospitalization to the use of the 3D printed titanium in surgery (including gathering and processing CT data of 1–2 days, 3D printing and processing for one day, heat treatment for one day, and polishing and surface anodizing for one day), a waiting time which was too long, resulting in increased pain for the patients. If the design, processing, and post-treatment could be optimized in the near future, with all of the resources concentrated geographically, the whole process could be reduced to two days and the waiting time for patients would, thus, be greatly decreased.

#### 4.4.3. Clinical Applications of the Customized Bone Plate after Improvement

**Figure 10:**
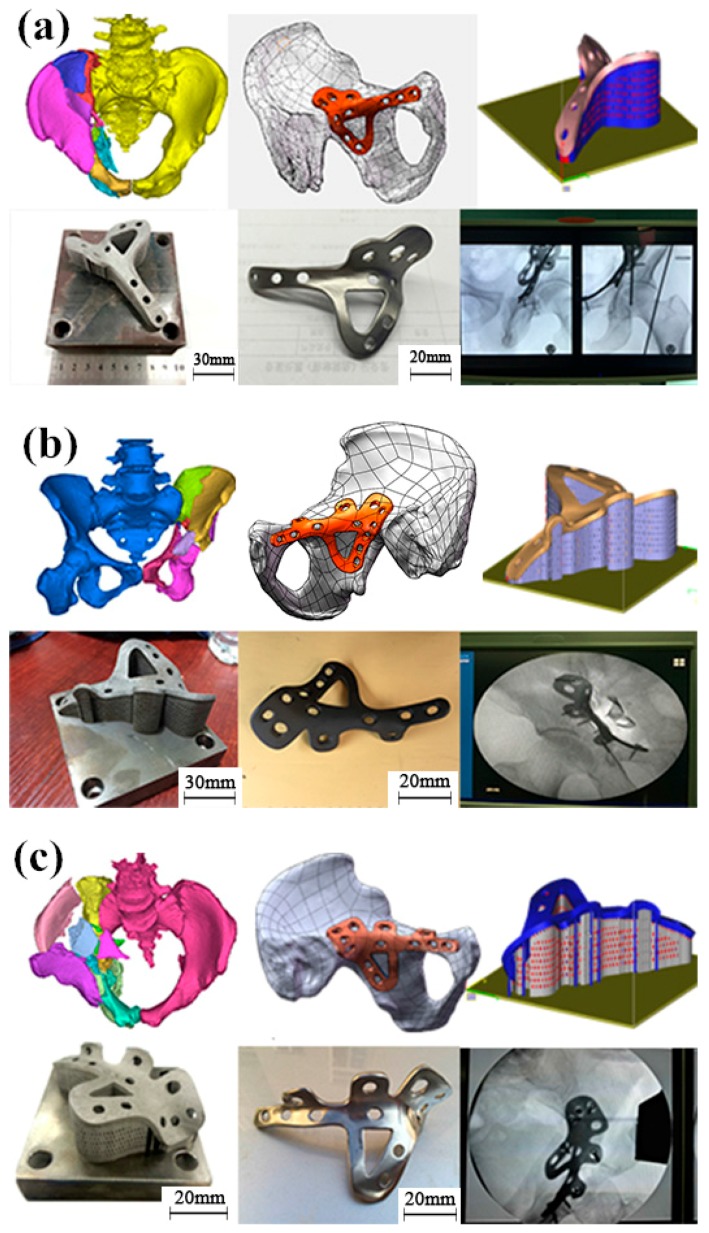
The other three successive clinical cases application process from design, metal 3D printing to clinical operation. (**a**) The second case; (**b**) The third case; (**c**) The fourth case.

According to the description above, that is, the design and fabrication processes of the customized bone plate for a complex pelvic fracture, which was achieved by metal 3D printing technology, three successful operations were successively performed in the present study, as presented in Figure 10.

Compared to the traditional technology of bone plate surgery, the use of a 3D-printed titanium alloy bone plate satisfied the complex requirements of different patients suffering pelvic fracture. By using the proposed technique, patients with similar symptoms could be treated with this accurate bone plate. The main difference is the customized bone plate design (the doctor is critical to the design), as each patient’s fracture position has a unique contact surface. Other than that, all other procedures should be the same. Future enhancements of the bone plate requires optimization of each stage, from design to clinical operation. The bone plate’s design requires communication between clinicians, designers, and SLM process engineers, in order that this technique can be used widely. As anatomical landmarks are not easily defined, the doctor’s clinical experience is always very important for the design.

## 5. Conclusions

In the present study, a customized bone plate was fabricated through customized design and metal 3D printing to satisfy the complex requirements of different patients suffering pelvic fracture.

1. Key procedures in designing customized bone plates included: (1) extraction of the CT data of the pelvis; (2) generation of a pelvic model; (3) fabrication of the initial shape of the bone plate; (4) determination of the shape and thickness of the bone plate; (5) generating of the screw holes; and (6) printing of the final bone plate model.
2. In order to guarantee the surface quality of the attaching face between bone plate and bone, the attaching surface of the plate was oriented upwards and a cone-type support added to the lower surface to improve heat conductivity during the SLM fabricating process. Heat treatment in vacuum was employed to eliminate residual stress to prevent the bone plate from deforming after fabrication. Following heat treatment the plate had a hardness of HV1 360–390, an ultimate tensile strength of 1000–1100 MPa, yield strength of 900–950 MPa, and an elongation of 8%–10%, thus satisfying operating requirements.
3. The customized bone plate was used in a clinical operation. Matching of the customized bone plate and broken pelvis was confirmed by intra operative C-arm and X-ray imaging following surgery, which not only fixed the broken bone blocks, but also guided pelvis restoration. Operating time was reduced to about 2 h.

## Figures and Tables

**Table 1:** Comparison between compositions of SLM fabricating powder and standard powder (part of the composition data obtained from reference [22]).

Element	Composition (%)	ASTMF2924 (%) [22]	Element	Composition (%)	ASTMF2924 (%) [22]
Al	6.0	5.50–6.75	N	0.012	<0.05
V	3.90	3.50–4.50	H	0.0022	<0.015
Fe	0.044	<0.3	Y	0	<0.005
O	0.10	<0.2	Ti	Balance	Balance
C	0.013	<0.08	other	<0.03	<0.4

**Table 2:** The optimized SLM fabricating parameters.

Laser Power (W)	Scanning Speed (mm/s)	Scanning Space (µm)	Layer Thickness (µm)	Spot Diameter (µm)
150	600	80	40	70

**Table 3:** Mechanical properties of 3D printed part.

Comparision Items	Ultimate Tensile Strength (MPa)	Yield Strength (MPa)	Elongation (%)	Hardness (HV1)
3D printing (before heat treatment)	1288.70 ± 6.44	1063.99 ± 5.32	6.43 ± 0.03	373 ± 1.9
3D printing (after heat treatment)	1081.42 ± 5.41	925.26 ± 4.63	8.11 ± 0.04	367 ± 1.9
standard cast part [22]	>895	>825	>6	320

## References

[1] Lund T., Laine T., Österman H., Yrjönen T., Schlenzka D. (2012). Accuracy of computer assisted pedicle screw insertion: The evidence. J. Bone Jt. Surg. Br..

[2] Ryken T.C., Owen B.D., Christensen G.E., Reinhardt J.M. (2009). Image-based drill templates for cervical pedicle screw placement. J. Neurosurg. Spine.

[3] Melkent T., Foley K.T., Estes B.T., Chaudoin J. (2002). Image Guided Spinal Surgery Guide, System, and Method for Use Thereof. U.S. Patent.

[4] Malik H.H., Darwood A.R., Shaunak S., Kulatilake P., El-Hilly A.A., Mulki O., Baskaradas A. (2015). Three-dimensional printing in surgery: A review of current surgical applications. J. Surg. Res..

[5] Martelli N., Serrano C., van den Brink H., Pineau J., Prognon P., Borget I., El Batti S. (2016). Advantages and disadvantages of 3-dimensional printing in surgery: A systematic review. Surgery.

[6] Condino S., Carbone M., Ferrari V., Faggioni L., Peri A., Ferrari M., Mosca F. (2011). How to build patient-specific synthetic abdominal anatomies. An innovative approach from physical toward hybrid surgical simulators. Int. J. Med. Robot. Comput. Assist. Surg..

[7] Tam M.D., Laycock S.D., Bell D., Chojnowski A. (2012). 3-D printout of a DICOM file to aid surgical planning in a 6 year old patient with a large scapular osteochondroma complicating congenital diaphysealaclasia. J. Radiol. Case Rep..

[8] Birnbaum K., Schkommodau E., Decker N., Prescher A., Klapper U., Radermacher K. (2001). Computer-assisted orthopedic surgery with individual templates and comparison to conventional operation method. Spine.

[9] Engel M., Bodem J.P., Hoffmann J., Freudlsperger C. (2012). Reconstruction of a near-total nasal defect using a precontoured titanium mesh with a converse scalping flap. J. Craniofac. Surg..

[10] Sugimoto M., Lee S.Y., Sakai Y., Nishida K., Kuroda R., Kurosaka M. (2014). Tactile surgical navigation system for complex acetabular fracture surgery. Orthopedics.

[11] Harrysson O.L.A., Marcellin-Little D.J., Horn T.J. (2015). Applications of metal additive manufacturing in veterinary orthopedic surgery. JOM.

[12] Sing S.L., An J., Yeong W.Y., Wiria F.E. (2016). Laser and electron-beam powder-bed additive manufacturing of metallic implants: A review on processes, materials and designs. J. Orthop. Res..

[13] Bartolo P., Kruth J.P., Silva J., Levy G., Malshe A., Rajurkar K., Leu M. (2012). Biomedical production of implants by additive electro-chemical and physical processes. CIRP Ann.-Manuf. Technol..

[14] Laird A., Keating J.F. (2005). Acetabular fractures: A 16-year prospective epidemiological study. J. Bone Jt. Surg. Br..

[15] Madhu R., Kotnis R., Al-Mousawi A., Barlow N., Deo S., Worlock P., Willett K. (2006). Outcome of surgery for reconstructionof fractures of the acetabulum- the time dependent effect of delay. J. Bone Jt. Surg. Br..

[16] Letournel E. (1993). The treatment of acetabular fractures through the ilioinguinalapproach. Clin. Orthop. Relat. Res..

[17] Matta J.M. (1994). Operative treatment of acetabular fractures through the Ilioinguinal approach a 10-year perspective. Clin. Orthop. Relat. Res..

[18] Kinik H., Armangil M. (2004). Extensile triradiate approach in the management of combined acetabular fractures. Arch. Orthop. Trauma Surg..

[19] Gong X., Anderson T., Chou K. Review on powder-based electron beam additive manufacturing technology. Proceedings of the ASME/ISCIE 2012 International Symposium on Flexible Automation.

[20] Do D.K., Li P. (2016). The effect of laser energy input on the microstructure, physical and mechanical properties of Ti-6Al-4V alloys by selective laser melting. Virtual Phys. Prototyp..

[21] Yadroitsev I., Yadroitsava I. (2015). Evaluation of residual stress in stainless steel 316L and Ti6Al4V samples produced by selective laser melting. Virtual Phys. Prototyp..

[22] (2014). Standard Specification for Additive Manufacturing Titanium-6 Aluminum-4 Vanadium with Powder Bed Fusion.

[23] Vrancken B., Thijs L., Kruth J.P., van Humbeeck J. (2014). Microstructure and mechanical properties of a novel β titanium metallic composite by selective laser melting. Acta Mater..

[24] Sing S.L., Yeong W.Y., Wiria F.E. (2016). Selective laser melting of titanium alloy with 50 wt % tantalum: Microstructure and mechanical properties. J. Alloys Compd..

[25] (2003). Standard Specification for Wrought Titanium-6Aluminum-4Vanadium ELI (Extra Low Interstitial) Alloy for Surgical Implant Applications.

[26] Basalah A., Shanjani Y., Esmaeili S., Toyserkani E. (2012). Characterizations of additive manufactured porous titanium implants. J. Biomed. Mater. Res. Part B.

[27] Gu D., Hagedorn Y.C., Meiners W., Meng G., Batista R.J.S., Wissenbach K., Poprawe R. (2012). Densification behavior, microstructure evolution, and wear performance of selective laser melting processed commercially pure titanium. Acta Mater..

